# Perspectives on validation of clinical predictive algorithms

**DOI:** 10.1038/s41746-023-00832-9

**Published:** 2023-05-06

**Authors:** Anne A. H. de Hond, Vaibhavi B. Shah, Ilse M. J. Kant, Ben Van Calster, Ewout W. Steyerberg, Tina Hernandez-Boussard

**Affiliations:** 1 Clinical AI Implementation and Research Lab, Leiden University Medical Centre, Leiden, the Netherlands; 2 Department of Medicine (Biomedical Informatics), Stanford University, Stanford, CA USA; 3 Department of Biomedical Data Sciences, Leiden University Medical Centre, Leiden, the Netherlands; 4 Department of Digital Health, University Medical Center Utrecht, Utrecht, the Netherlands; 5 Department of Development & Regeneration, KU Leuven, Leuven, Belgium; 6 Department of Biomedical Data Science, Stanford University, Stanford, CA USA; 7 Department of Epidemiology & Population Health (by courtesy), Stanford University, Stanford, CA USA

**Keywords:** Epidemiology, Machine learning, Bioinformatics, Statistical methods

## Abstract

The generalizability of predictive algorithms is of key relevance to application in clinical practice. We provide an overview of three types of generalizability, based on existing literature: temporal, geographical, and domain generalizability. These generalizability types are linked to their associated goals, methodology, and stakeholders.

Machine learning has led to a surge in the development of clinical predictive algorithms. The generalizability of these algorithms often goes untested^1^, leaving the community in the dark on their accuracy and safety when applied to a specific medical setting. We need clear objectives with respect to generalizability that align with the intended use. Journals, funding organizations, and regulatory bodies provide some guidance on generalizability requirements for clinical predictive algorithms, but a clear definition is often lacking. For example, it is considered best practice to ‘Describe the generalizability of the model including the performance of the model on validation and testing datasets^2^’. We consider this recommendation too vague. It is not clear what type of generalizability is referred to and whether it is sufficient for the intended use of the algorithm (see Supplementary Table 1 for more examples and suggestions for improvement). This commentary aims to provide clarity on different objectives related to generalizability via an overview of three main types of generalizability summarized from the literature with their associated goals, methodology, and stakeholders.

We performed a scoping review to identify different types of generalizability (see Supplementary Methods and Supplementary Table 2). In the context of clinical prediction models or predictive algorithms, generalizability refers to an algorithm’s ability to perform adequately across different settings^3^. Setting is defined by the clinical context of included patients, time, and place. Algorithm performance can then be assessed along various axes, including discrimination^3^, calibration^4^, and measures for clinical usefulness, such as Net Benefit^5^. We extracted three distinct types of generalizability. Examples of published validation use cases for each generalizability type can be found in Supplementary Table 3.

A key distinction should be made between internal and external validation (Fig. 1). Internal validation assesses the reproducibility of algorithm performance in data that is distinct from the development (or: train) data but derived from the exact same underlying population. It provides an optimism-corrected estimate of performance for the setting where the data originated from^6^. Cross-validation and bootstrapping are the recommended methods to assess internal validity^6,7^. Cross-validation splits the data in equal parts (usually five or ten) and trains the algorithm on all but one holdout part that is used for testing. This process is repeated until all parts have been used as test data. The whole procedure is preferably repeated multiple times for more stability, e.g., a 10 × 10-fold cross-validation procedure. Bootstrapping repeatedly samples data points from the development data with replacement (usually 500–2000 times). These samples are used to train the algorithm with the original development data as test set^6,8^. Internal validation is necessary but not sufficient to ensure safe clinical applicability. The main stakeholder is the developer of the algorithm, who uses internal validation to assess the validity of the development process, and quantifies overoptimism in expected performance^7,9^.

**Fig. 1:**
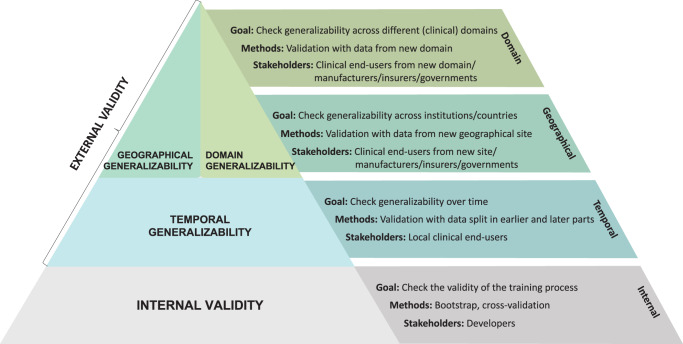
Generalizability types. Schematic overview of the different types of generalizability with the validation’s goals, methods, and stakeholders.

External validity assesses the transportability of the clinical predictive algorithm to other settings than those considered during development (Fig. 1). It encompasses three generalizability types: temporal, geographical and domain generalizability. Temporal validity assesses the performance of an algorithm over time at the development setting. This type of generalizability is required to understand data drift (a change in the data over time from the data that was used during development)^10^. Temporal validity may be assessed by testing the algorithm on a dataset derived from the same setting as the development cohort but from a later time. Variations in design are possible, such as a ‘waterfall’ design, in which the development time window is repeatedly increased^11^. The main stakeholders of temporal validity are clinicians, hospital administrators, and other clinical end-users that plan to implement the algorithm into their clinical practice. These stakeholders need proof of temporal validity to ensure the safe use of the algorithm at their local clinical institution or hospital.

Geographical validation assesses the generalizability of an algorithm to a place (institution or location) that is different from where the algorithm was developed. This type of validation assesses the heterogeneity across places. Geographical validity can be assessed by testing the algorithm on data collected from the new place(s). More complex designs are possible, such as a leave-one-site-out (or internal-external) validation in which the algorithm is developed on all but one location and tested on the left-out one^12^. This process is repeated until all locations have been used as test location. Geographical validation is required when the algorithm is going to be used outside of the original development place. The main stakeholders are the clinical end-users at a new implementation site who want proof of validity for safe use at their site. Manufacturers, insurers, and governing bodies could be other stakeholders that are interested in evidence for the general or widespread applicability of the prediction tool. When geographical generalizability is low, a global model that is valid for different places may not be tenable^13^. Instead, a local variant of the algorithm could be achieved through updating the global algorithm at each individual place^4^.

Domain validation assesses the generalizability of an algorithm to a different clinical context^14,15^. This type of validation considers generalizability across medical background (e.g., 30-day mortality risk for emergency versus surgical patients), but also medical setting (e.g., fall prevention in nursing home versus hospital), and demographics (e.g., emergency admission risk for adult versus pediatric patients). For example, some COVID-19 prediction models were developed for related respiratory diagnoses^16^. In a large study on generalizability of prediction models, model performance was found to be better in ‘closely related’ than ‘distantly related’ validation cohorts, which underscores the relevance of domain generalizability^17^. Like geographical validation, domain validity is assessed by testing the algorithm on data collected from a new domain. Stakeholders of domain validity include clinical end-users from the new domain, manufacturers, insurers, and governing bodies. If the algorithm does not generalize across domains, the underlying relationships may be truly different, warranting separate algorithms for each domain.

The overview presented in Fig. 1 may be used as a starting point by regulatory bodies, industry, and academia when formulating guidelines and requirements for the generalizability of a clinical predictive algorithm. Building on previous work^18–21^, we argue that validation studies should be suited to the target context and the intended use of the clinical predictive algorithm. Always aiming for a specific type of generalizability may not be defensible for some predictive algorithms and their intended use^18,22^.

During algorithm development and validation, researchers and developers should adhere to guidelines, specifically TRIPOD or its forthcoming variant, TRIPOD-AI^20,23^. They should report on the algorithm’s capacity to generalize and provide a justification for their chosen validation strategy by relating it to their intended operational period, (clinical) population, and environment. Moreover, they should add a disclaimer about the type of generalizability and intended use of their algorithms. If generalizability is limited this ought to be acknowledged alongside other implementation risks. For example, only internal or temporal validation was performed, or poor generalizability was found across places or clinical contexts. Researchers and developers should also report when the algorithm’s scope limits the necessary validation steps. For example, domain validation may not be attempted when a predictive algorithm cannot be used (or has very limited use) outside of its domain (e.g., a prostate biopsy model).

In conclusion, we propose more precise specification for the desired and required type of generalizability for the implementation of clinical predictive algorithms. The three generalizability types discussed here, comprising temporal, geographical, and domain generalizability, all serve a unique goal and specific application purpose. Hence, researchers, developers, journals, funding organizations, and regulatory bodies should ensure that their chosen generalizability claims on the algorithm’s intended use align with the underlying evidence. Future research may assess the impact of different types of heterogeneity on generalizability and steps to improve generalizability for clinical predictive algorithms.

## Supplementary information

Supplemental Information
