# Supplementary material for: Perspectives on validation of clinical predictive algorithms
Source: NPJ Digit Med. 2023 May 6;6:86. doi: 10.1038/s41746-023-00832-9 (PMC10163568; doi:10.1038/s41746-023-00832-9)
Supplement: Supplementary file 1 — Supplemental Information [file 41746_2023_832_MOESM1_ESM.pdf]

Supplemental Material

Perspectives on validation of clinical predictive algorithms

Anne A.H. de Hond, Vaibhavi B. Shah, Ilse M.J. Kant, Ben Van Calster, Ewout W. Steyerberg, Tina Hernandez-Boussard

Supplementary Tables

Supplementary Table 1. Historic recommendations on generalizability and validation from academia, regulatory bodies, and funding agencies

Source	Statements on generalizability and validation	Suggestions for improvement
Academia	"Define the model validation strategies. Internal validation is the minimum requirement; external validation should also be performed whenever possible." [1]	In this paper by Luo et al., it is ambiguous what is meant by 'external validation'. It is not clear what type of generalizability is referred to here and whether it aligns with the intended use.<br>Suggestion: 'Define the model validation strategies and ensure that they are aligned with the intended operational period, (clinical) population and environment…'.
Academia	"Describe the generalizability of the model, including the performance of the model on validation and testing datasets." [2]	The 'generalizability of the model' is too vague in these otherwise excellent best practices by Kakarmath et al. It is not clear what type of generalizability is meant here and whether it suffices for the intended use.<br>Suggestion: 'Describe the generalizability of the model to the intended setting, including operational period, (clinical) population and environment…'.
Regulatory body	"Did you put in place verification and validation methods and documentation (e.g., logging) to evaluate and ensure different aspects of the AI system's reliability and reproducibility? Did you clearly document and operationalize processes for the testing and verification of the reliability and reproducibility of the AI system?" [3]	In this document by the High-Level Expert Group on Artificial Intelligence, 'reproducibility' may refer to internal validity, but this could be made explicit. 'Validation methods' are too vague. It is not clear if this only refers to internal validity or also extends to external validity, for example temporal validity.<br>Suggestion: 'Did you put in place internal validation methods and documentation (e.g., logging) to evaluate and ensure different aspects of the AI system's reliability and reproducibility? Did you clearly document and operationalize processes for the testing and verification of the internal validity (and possibly temporal validity) of the AI system?'.
Regulatory body	"The product must then be validated which usually involves being tested in a setting that represents the intended population and/or environment." [4]	The UK Department for Health and Social Care correctly states that the validation should be aligned with the intended use of the predictive algorithm. It could be further improved by phrasing this more strictly as a best practice.<br>Suggestion: 'The product must then be validated in a setting that represents the intended operational period, (clinical) population and environment'.

Regulatory body	"Analytical validation confirms and provides objective evidence that the software was correctly constructed – namely, correctly and reliably processes input data and generates output data with the appropriate level of accuracy, and repeatability and reproducibility … Analytical validation is necessary for any SaMD." [5]	It is unclear what is referred to by 'analytical validation' in this FDA document. From the context, it appears to refer to internal validation ('reproducibility').<br><br>Suggestion: 'Internal validation confirms and provides objective evidence that the software was correctly constructed – namely, correctly and reliably processes input data and generates output data with the appropriate level of accuracy, and repeatability and reproducibility… Internal validation is necessary for any SaMD'.
Regulatory body	Annex I: "17.1. Devices that incorporate electronic programmable systems, including software, or software that are devices in themselves, shall be designed to ensure repeatability, reliability and performance in line with their intended use… 17.2. For devices that incorporate software or for software that are devices in themselves, the software shall be developed and manufactured in accordance with the state of the art taking into account the principles of development life cycle, risk management, including information security, verification and validation. " [6]	The first part of this statement from the Medical Device Regulation addresses internal validity ('reliability', 'repeatability') in line with the intended use of the software. The second part is vague, stating that the 'principles of … validation' should be considered.<br><br>Suggestion: '17.1 Devices that incorporate electronic programmable systems, including software,… shall be designed to ensure internal validity and performance in line with their intended use… the software shall be developed and manufactured in accordance with the state of the art taking into account the principles of development life cycle, risk management, including information security, verification and validation that is performed on the intended operational period, (clinical) population and environment'.
Funding agency	"Applicants should clearly justify the importance and implications of validating digital health and AI tools and technologies. Studies should apply rigorous research methods to evaluate the effectiveness of any proposed digital health and AI applications including the use of gold-standard comparators." [7]	In this call for proposals, the National Institute of Health stresses the importance of validation of AI tools. It is not clear what type of validation they require and that it should align with the intended use.<br><br>Suggestion: 'Applicants should clearly justify the importance and implications of validating digital health and AI tools and technologies in their intended operational period, (clinical) population and environment'.

Supplementary Table 2. Overview of articles included in the scoping review

Authors	Article Title	Year	Journal
Justice, Covinsky, & Berlin	Assessing the generalizability of prognostic information.	1999	*Annals of Internal Medicine*
Altman & Royston	What do we mean by validating a prognostic model?	2000	*Statistics in Medicine*
Wade	Derivation versus validation.	2000	*Archives of Disease Childhood*
Toll, Janssen, Vergouwe, & Moons	Validation, updating and impact of clinical prediction rules: a review.	2008	*Journal of Clinical Epidemiology*
Altman, Vergouwe, Royston, & Moons	Prognosis and prognostic research: validating a prognostic model.	2009	*BMJ*
Halabi & Owzar	The importance of identifying and validating prognostic factors in oncology.	2010	*Seminars in Oncology*
Mallett, Royston, Waters, Dutton, & Altman	Reporting performance of prognostic models in cancer: a review.	2010	BMC Medicine
Vickers	Prediction models in cancer care.	2011	*CA: A Cancer Journal for Clinicians*
Bouwmeester, Zuithoff, Mallett, Geerlings, Vergouwe, Steyerberg, et al.	Reporting and Methods in Clinical Prediction Research: A Systematic Review.	2012	*Plos Medicine*
Moons, Kengne, Grobbee, Royston, Vergouwe, Altman, & Woodward	Risk prediction models: II. External validation, model updating, and impact assessment.	2012	Heart
Steyerberg, Moons, van der Windt, Hayden, Perel, Schroter, et al.	Prognosis Research Strategy (PROGRESS) 3: Prognostic Model Research.	2013	*Plos Medicine*
Collins, de Groot, Dutton, Omar, Shanyinde, Tajar, et al.	External validation of multivariable prediction models: a systematic review of methodological conduct and reporting.	2014	*BMC Medical Research Methodology*
Labarère, Renaud, & Fine	How to derive and validate clinical prediction models for use in intensive care medicine.	2014	*Intensive Care Medicine*
Steyerberg & Vergouwe	Towards better clinical prediction models: seven steps for development and an ABCD for validation.	2014	*European Heart Journal*
Collins, Reitsma, Altman, & Moons	Transparent reporting of a multivariable prediction model for individual prognosis or diagnosis (TRIPOD): the TRIPOD Statement.	2015	*BMC Medicine*
Debray, Vergouwe, Koffijberg, Nieboer, Steyerberg, & Moons	A new framework to enhance the interpretation of external validation studies of clinical prediction models.	2015	*Journal of Clinical Epidemiology*
Dimitrov, Motterlini, & Fahey	A simplified approach to the pooled analysis of calibration of clinical prediction rules for systematic reviews of validation studies.	2015	*Clinical Epidemiology*
Austin, van Klaveren, Vergouwe, Nieboer, Lee, & Steyerberg	Geographic and temporal validity of prediction models: different approaches were useful to examine model performance.	2016	*Journal of Clinical Epidemiology*
Lee, Bang, & Kim	How to Establish Clinical Prediction Models.	2016	Endocrinology and Metabolism
Mansmann, Rieger, Strahwald, & Crispin	Risk calculators-methods, development, implementation, and validation.	2016	International Journal of Colorectal Disease

Nieboer, van der Ploeg, & Steyerberg	Assessing Discriminative Performance at External Validation of Clinical Prediction Models.	2016	*PLoS One*
Snell, Hua, Debray, Ensor, Look, Moons, & Riley	Multivariate meta-analysis of individual participant data helped externally validate the performance and implementation of a prediction model.	2016	*Jounral of Clinical Epidemiology*
Cowley, Farewell, Maguire, & Kemp	Methodological standards for the development and evaluation of clinical prediction rules: a review of the literature.	2019	*Diagnostic and Prognostic Research*
Burns & Kheterpal	Machine Learning Comes of Age: Local Impact versus National Generalizability.	2020	*Anesthesiology*
Futoma, Simons, Panch, Doshi-Velez, & Celi	The myth of generalisability in clinical research and machine learning in health care.	2020	*The Lancet Digital Health*
Hernandez-Boussard, Bozkurt, Ioannidis, & Shah	MINIMAR (MINimum Information for Medical AI Reporting): Developing reporting standards for artificial intelligence in health care.	2020	*Journal of the American Medical Informatics Association*
Ho, Phua, Wong, & Bin Goh	Extensions of the External Validation for Checking Learned Model Interpretability and Generalizability.	2020	*Patterns*
Leisman, Harhay, Lederer, Abramson, Adjei, Bakker, et al.	Development and Reporting of Prediction Models: Guidance for Authors From Editors of Respiratory, Sleep, and Critical Care Journals.	2020	Critical Care Medicine
Azad, Ehresman, Ahmed, Staartjes, Lubelski, Stienen, et al.	Fostering reproducibility and generalizability in machine learning for clinical prediction modeling in spine surgery.	2021	*The Spine Journal*
Nay & Strandburg	Generalizability: Machine Learning and Humans-in-the-Loop	2021	*Research handbook on big data law*.
Ramspek, Jager, Dekker, Zoccali, & van Diepen	External validation of prognostic models: what, why, how, when and where?	2021	*Clinical Kidney Journal*
de Hond, Leeuwenberg, Hooft, Kant, Nijman, van Os, et al.	Guidelines and quality criteria for artificial intelligence-based prediction models in healthcare: a scoping review.	2022	*NPJ Digital Medicine*
Wan, Caffo, & Vedula	A Unified Framework on Generalizability of Clinical Prediction Models.	2022	*Frontiers in Artificial Intelligence*

Supplementary Table 3. Examples of different generalizability types

**Type of validity/ generalizability**	**Example**
Internal validity	The 4C Mortality Score was developed in 260 hospitals across the United Kingdom and L1 penalized coefficients were derived using 10-fold cross validation [8].
	A machine learning model for predicting readmission or death within 7 days after ICU discharge was developed in one academic medical center in the Netherlands using 10-fold cross-validation [9].
Temporal generalizability	The 4C Mortality score was developed on a cohort recruited between 6 February and 20 May 2020 and temporally validated on a cohort recruited between 21 May and 29 June 2020 [8].
	A machine learning model for predicting readmission or death within 7 days after ICU discharge was developed on a cohort recruited between 2004 and March 2016 and temporally validated on a cohort recruited between March 2016 and 2019 [9].
Geographical generalizability	The 4C Mortality Score was externally validated in a Canadian and Japanese cohort with different geography from the development cohort (United Kingdom) [10, 11].
	A sepsis prediction model was externally validated in an academic health system with different geography from the development cohort [12].
	A machine learning model for predicting readmission or death within 7 days after ICU discharge was externally validated in an academic medical center (Leiden University Medical Center) with different geography from the development center (Amsterdam Medical Center, Location VUmc) [13].
Domain generalizability	An emergency medicine admissions model was developed in two urban teaching centers and one tertiary care center in the Netherlands. It was validated by a leave one group out cross-validation where each center formed one group to address the heterogeneity between the different hospital types [14].
	Three ED disposition prediction model were developed. Two were developed in Children hospital's EDs and one in a community general hospital's ED. These models were than validated on the other two sites [15].

Supplementary Methods

We performed a scoping review to identify different types of generalizability. We did not aim for a comprehensive list of all types of generalizability, but rather for an overview of the most often mentioned generalizability objectives and validation methods for prediction models and machine learning. In Pubmed, we screened papers that were returned by the following searches (August 11, 2022): "((generalizable[Title]) OR (generalisable[Title]) OR (generalizability[Title]) OR (generalisability[Title])) AND ((model[Title]) OR (machine learning[Title]) OR (artificial intelligence[Title]))" (106 papers). In Google Scholar, we screened papers that were returned by the following searches (August 11, 2022): “allintitle: (generalizable OR generalisable OR generalizability OR generalisability) AND (machine learning)” (38 papers); “allintitle: (generalizable OR generalisable OR generalizability OR generalisability) AND (artificial intelligence)” (11 papers); “allintitle: (generalizable OR generalisable OR generalizability OR generalisability) AND (model)” (154 papers). Snowballing of references was applied for relevant papers, with a focus on papers published in the last 10 years.

Supplementary References

1. Luo, W., D. Phung, T. Tran, et al., *Guidelines for Developing and Reporting Machine Learning Predictive Models in Biomedical Research: A Multidisciplinary View.* Journal of Medical Internet Research, 2016. **18**(12).
2. Kakarmath, S., A. Esteva, R. Arnaout, et al., *Best practices for authors of healthcare-related artificial intelligence manuscripts.* npj Digital Medicine, 2020. **3**(1): p. 134.
3. High-Level Expert Group on Artificial Intelligence, *Assessment List for Trustworthy Artificial Intelligence (ALTAI) for self-assessment.* 2020.
4. Department of Health & Social Care, *A guide to good practice for digital and data-driven health technologies.* 2021.
5. FDA, *Software as a Medical Device (SAMD): Clinical Evaluation.* 2017.
6. Council of the European Union, *Regulation (EU) 2017/745 of the European Parliament and of the Council of 5 April 2017 on medical devices, amending Directive 2001/83/EC, Regulation (EC) No 178/2002 and Regulation (EC) No 1223/2009 and repealing Council Directives 90/385/EEC and 93/42/EEC (Medical Device Regulation).* 2017.
7. National Institute of Health (NIH). *Notice of Special Interest (NOSI): Validation of Digital Health and Artificial Intelligence Tools for Improved Assessment in Epidemiological, Clinical, and Intervention Research*. 2022; Available from: https://grants.nih.gov/grants/guide/notice-files/NOT-CA-22-037.html
8. Knight, S.R., A. Ho, R. Pius, et al., *Risk stratification of patients admitted to hospital with covid-19 using the ISARIC WHO Clinical Characterisation Protocol: development and validation of the 4C Mortality Score.* BMJ, 2020. **370**: p. m3339.
9. Thoral, P.J., M. Fornasa, D.P. de Bruin, et al., *Explainable Machine Learning on AmsterdamUMCdb for ICU Discharge Decision Support: Uniting Intensivists and Data Scientists.* Crit Care Explor, 2021. **3**(9): p. e0529.
10. Jones, A., T. Pitre, M. Junek, et al., *External validation of the 4C mortality score among COVID-19 patients admitted to hospital in Ontario, Canada: a retrospective study.* Scientific Reports, 2021. **11**(1): p. 18638.
11. Kuroda, S., S. Matsumoto, T. Sano, et al., *External validation of the 4C Mortality Score for patients with COVID-19 and pre-existing cardiovascular diseases/risk factors.* BMJ Open, 2021. **11**(9): p. e052708.
12. Wong, A., E. Otles, J.P. Donnelly, et al., *External Validation of a Widely Implemented Proprietary Sepsis Prediction Model in Hospitalized Patients.* JAMA Internal Medicine, 2021. **181**(8): p. 1065-1070.

13. de Hond, A.A.H., I.M.J. Kant, M. Fornasa, et al., *Predicting Readmission or Death After Discharge From the ICU: External Validation and Retraining of a Machine Learning Model.* Critical Care Medicine, 2023. **51**(2).
14. De Hond, A., W. Raven, L. Schinkelshoek, et al., *Machine learning for developing a prediction model of hospital admission of emergency department patients: Hype or hope?* Int J Med Inform, 2021. **152**: p. 104496.
15. Barak-Corren, Y., P. Chaudhari, J. Perniciaro, M. Waltzman, A.M. Fine, and B.Y. Reis, *Prediction across healthcare settings: a case study in predicting emergency department disposition.* npj Digital Medicine, 2021. **4**(1): p. 169.
